# How to gain evidence for causation in disease and therapeutic intervention: from Koch’s postulates to counter-counterfactuals

**DOI:** 10.1007/s11019-022-10096-x

**Published:** 2022-07-06

**Authors:** David W. Evans

**Affiliations:** 1 Centre of Precision Rehabilitation for Spinal Pain, School of Sport, Exercise and Rehabilitation Sciences, University of Birmingham, B15 2TT Edgbaston, Birmingham, UK; 2 Research Centre, University College of Osteopathy, London, UK

## Abstract

Researchers, clinicians, and patients have good reasons for wanting answers to causal questions of disease and therapeutic intervention. This paper uses microbiologist Robert Koch’s pioneering work and famous postulates to extrapolate a logical sequence of evidence for confirming the causes of disease: *association* between individuals with and without a disease; *isolation* of causal agents; and the creation of a *counterfactual* (demonstrating that an agent is sufficient to reproduce the disease anew). This paper formally introduces *counter-counterfactuals*, which appear to have been used, perhaps intuitively, since the time of Koch and possibly earlier. An argument is presented that counter-counterfactuals (disease-preventers) are a useful tool for identifying necessary causes of disease, and sometimes must be used in place of isolation which is not always possible. In addition, a logical sequence of causal evidence for a therapeutic intervention is presented: creating a *counterfactual* (demonstrating that the intervention is sufficient to change the natural course of a disease), *comparisons* between subjects in receipt of treatment versus those who are not (typically within a randomised controlled trial, which can quantify effects of intervention), and *counter-counterfactuals* (treatment-preventers, which can identify the intervention’s mechanisms of action).

## Introduction

Researchers, clinicians, and patients have good reasons for wanting answers to causal questions relating to disease and therapeutic intervention. For example, “How does factor *x* cause disease *y* in a formerly healthy person?” and “How does treatment *z* improve disease *y*?” To answer these important causal questions, two categories of evidence are required: (1) evidence for disease causation, and (2) evidence for the causal action(s) of an intervention. This paper discusses these two categories in turn. For each category, a logical sequence of evidence is presented, through which causal claims may be justified. It is hoped that an explicit description of these sequences will be useful to the research community when planning future studies, to clinicians when making difficult decisions with an incomplete evidence base, and to patients for better understanding their ailments and treatments.

## Part one: evidence for disease causation

**Fig. 1:**
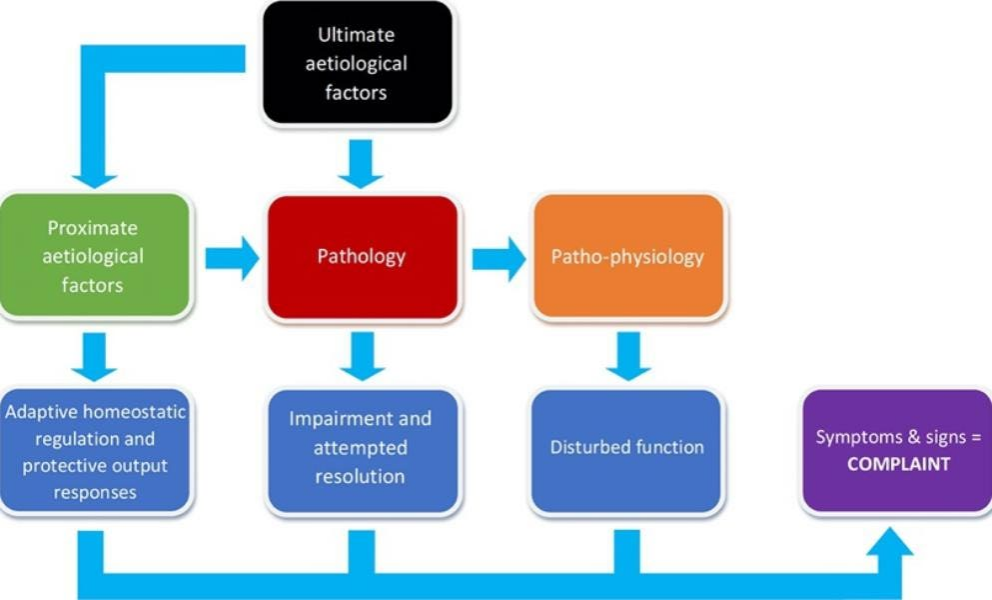
The pathological model of disease

The most enduring model of disease causation (i.e., how a disease begins, develops, and acts within an organism) is the pathological model (Fig. 1). The model describes the chronological progression through stages of a disease; aetiological factors collectively contribute to the emergence of an altered state (pathology, or pathosis) within one or more organ systems, which in turn can create and maintain disturbances in function, or ‘pathophysiology’ (Bogduk 2001). As its name suggests, pathology is of central importance to this model of disease. The model is asymmetrical in that diseases begin and progress in one direction. One stage cannot begin until the previous stage has begun, with each stage of the model capable of continuing despite commencement of the next.

In the nineteenth century, the ‘germ theory’ provided the first truly successful causal explanation for agents of infectious disease. In turn, these discoveries led to the mass-production of highly successful interventions (such as antibiotics), saving many millions of lives (Hutchings et al. 2019). Since the germ theory was based on causal hypotheses, research to support it required methodologies capable of testing and upholding causal claims. Accordingly, pioneering German physician, Robert Koch, building upon the work of Friedrich Loeffler, Edwin Klebs, and particularly Jakob Henle (Evans 1976; Kaufmann and Schaible 2005), developed a methodological blueprint for producing a chain of evidence to implicate microbes (or ‘parasites’ as they were sometimes referred to) as the necessary and sufficient causal agents of certain infectious diseases. His famous postulates (Table 1) are captured within a single paragraph (Koch 1891):

“*Wenn es sich nun aber nachweisen liess: erstens, dass der Parasit in jedem einzelnen Falle der betreffenden Krankheit anzutreffen ist, und zwar unter Verhältnissen, welche den pathologischen Veranderungen und dem klinischen Verlauf der Krankheit entsprechen: zweitens, dass er bei keiner anderen Krankheit als zufälliger und nicht pathogener Schmarotzer vorkommt; und drittens, dass er, von dem Körper vollkommen isolirt und in Reinculturen hinreichend oft umgezuchtet, im Stande ist, von Neuem die Krankheit zu erzeugen; dann konnte er nicht mehr zufalliges Accidens der Krankheit sein, sondern liess sich in diesem Falle kein anderes Verhältnis mehr zwischen Parasit und Krankheit denken, als dass der Parasit die Ursache der Krankheit ist*.”

Which can be translated to:

*“If, however, it can be proven: firstly, that the parasite is found in every single case of the disease in question, under circumstances which correspond to the pathological changes and the clinical course of the disease; secondly, that the parasite does not occur in any other disease as an incidental and non-pathogenic parasite; and thirdly, that, when completely isolated from the body and repeatedly propagated as a pure culture a sufficient number of times, the parasite can induce the disease anew; then it cannot be a coincidental accident of the disease, and in this case no other relationship between parasite and disease could be conceived than the parasite being the cause of the disease.”*

Although not a postulate of Koch, the first necessary step towards knowledge of disease causation must be disease characterisation (Thagard 1999). Symptoms, signs, pathological changes, and the natural course of a disease must be characterised as fully as possible so that the presence of the disease can be measured. After all, it is impossible to test either cause or effect if one cannot first measure them. Beyond this, Koch explicitly utilised three distinct forms of evidence for disease causation (Table 1): *association*, *isolation*, and *disease recreation*.

**Table 1:** Mechanistic evidence for disease causation, derived from Koch’s postulates

Koch’s postulates for microbial agents	General principle	Evidence for causation
The parasite is found in every single case of the disease in question, under circumstances which correspond to the pathological changes and the clinical course of the disease	Suspected causal agent(s) of a disease must be (or have previously been) present in every case of the disease	***Association: ubiquity***
The parasite does not occur in any other disease as an incidental and non-pathogenic parasite	Suspected causal agent(s) should not be (or have previously been) present in individuals who do not have (and have never had) the disease	***Association: exclusivity***
When completely isolated from the body and repeatedly propagated as a pure culture a sufficient number of times…	The suspected causal agent(s) must be isolated from all potential confounders	***Isolation***
… the parasite can induce the disease anew	Introducing the isolated agent(s) to a healthy subject, at an appropriate spatial location, should recreate the original disease	***Disease recreation***

### Association

The observation that a potential causal agent is consistently within, or at least spatially proximate to, subjects with a particular disease is usually the first evidence to arouse suspicion of a causal relationship (Cheng 1997). As such, Koch rightly stipulated that the same suspected causal agent must be, or have been, present in every naturally occurring case of a given disease. In other words, Koch considered specific germs to be *necessary* for specific diseases. He also knew that spatial co-incidence of a suspected causal agent and a diseased subject was insufficient to justify a claim of a causal relationship. Not only did Koch require evidence of the agent’s ubiquity in all subjects with the same disease, but he also expected its total exclusion from subjects free of the disease. These criteria represent the extreme ends of a prevalence spectrum: *all* or *nothing*.

Koch also believed that the presence of a specific pathogen would be sufficient for a specific disease. It is reasonable to expect that a necessary causal agent should not be (or have previously been) present in individuals who do not have (and have never had) the disease. Koch’s sound logic was unfortunately betrayed by Nature, however. He eventually had to abandon his demand for total exclusivity once he realised that carriers of a pathogen (e.g., *Vibrio cholerae*) could be asymptomatic. This discovery, that disease was not inevitable following exposure, was important because it weakened future arguments that exposure to certain causal agents was sufficient for given diseases.

To be clear, Koch considered causation to be deterministic (Gillies 2019); i.e., the causative mechanism was not probabilistic. However, the discovery of asymptomatic carriers of necessary causal agents in the population meant that the *evidence* demonstrating the presence or absence of these agents, in those with and without a disease, must always be probabilistic. Accordingly, 75 years after Koch’s postulates were formulated, the British epidemiologist, Sir Austin Bradford Hill, reminded us that consideration of ‘specificity of the association,’ when carefully applied, is a very useful tool when gathering evidence for disease aetiology (Hill 1965).

### Isolation

Successful isolation of a causal agent is arguably the most direct approach to discovering the cause of a disease. Once isolated and remote from any potential confounders, the structure and actions of a suspected causal agent can be more easily explored, tested and identified. In microbiology, isolation usually refers to the processes involved in obtaining a pure strain of a microorganism (or virus) from a mixed culture. Koch would first extract a tissue sample from a diseased host and from this attempt to grow a suspected pathogen on lifeless, cell-free media *in vitro*. He would then visually inspect his culture under the microscope to check that it was isomorphic and free from any host tissue. From a diseased subject, there would almost certainly be more than one potential causal agent available for extraction. Koch would therefore have to look for a candidate that was common to multiple subjects with the same disease. This extraction and inspection process would be repeated numerous times until he was confident that he had attained a pure culture (i.e., a single species of microbe). Once this was achieved, he could describe the characteristics of his specimen, interrogate the sample, and eventually introduce some of it to a healthy subject to see if it produced disease that was identical to that of the original host.

### Disease recreation

Koch knew that a disproportionately higher prevalence of a suspected causal agent in diseased subjects, compared to disease-free subjects, was insufficient to justify a strong claim of causality. At best, this finding only provides evidence for *spatial proximity* between cause and effect: necessary but not sufficient evidence for a causal claim. At worst, the suspected causal agent could just be a surrogate for the true cause (e.g., a by-product of the infection), thus constituting little more than a biomarker for a diagnostic test.

Although not explicitly mentioned within his postulates, Koch utilised control (comparison) subjects within his experiments. He would have several, nearly identical test subjects at his disposal (mice eventually became his preferred subjects, but rabbits and guinea pigs were regularly used). These test subjects would remain healthy (he knew this from comparisons with the unexposed control subjects) until he would inoculate them with a sample of his pure culture and observe whether the disease from the original host unfolded. This deliberate exposure is what Gillies (2016, 2019) describes as a ‘production action.’

Creating the disease anew at a time of one’s choosing also provides crucial evidence for *temporal precedence*: introduction of the pathogen temporally precedes the effect. Temporal precedence (or at the very least, simultaneity) is arguably the most fundamental requirement for a causal claim. Additionally, to recreate the original disease, Koch would first have to introduce the causal agent to a healthy subject at an appropriate spatial location. Indeed, several attempts over multiple test subjects may have been required to recreate the original disease, since some microbial agents can cause different diseases depending on their spatial presence (for instance, *Staphylococcus aureus* is capable of causing skin infections, pneumonia, heart valve infections, and bone infections).

Koch’s recreation of disease in specific healthy subjects of his choosing, and at a time decided by him, provides powerful causal evidence. After all, the ability to create a particular disease at will demonstrated that Koch’s sample was *sufficient* to produce the disease (Broadbent 2013). All required causal ingredients were present and accounted for, even if these ingredients remained unidentified at the time. He had intuitively constructed what philosophers call a *counterfactual* conditional. To understand a what a counterfactual conditional is, it helps to consider two separate events. As Scottish philosopher, David Hume proposed (Hume 1748), *if the first event had not been, the second would have never existed*. In other words, had Koch not inoculated a test subject with his isolated pathogen, the disease of interest would not have arisen in that subject. This is why naturally occurring cases of a disease do not qualify as counterfactuals; it is the *control* over both temporality and spatiality that makes a counterfactual so powerful. A counterfactual produces evidence of what philosophers call ‘difference-making’ (Russo & Williamson 2007, 2011). Koch’s systematic incorporation of counterfactuals within his experimental methodology is what sets his work apart from all prior investigations into the cause of disease (not just infectious disease). As Pearl and Mackenzie (2018) might say, Koch had climbed to the top rung of the ‘ladder of causation.’

### Vulnerabilities

Although Koch’s incorporation of counterfactuals within his methodology effectively secured his claims for causality, his isolation process – based as it was on multiple cycles of extraction, propagation, and visual inspection – contained some vulnerabilities. Koch’s isolation process worked for disease recreation because a bacterium is a sufficient cause of certain diseases. However, not all diseases are caused by a single causal agent. Furthermore, if only a portion of a microbe was extracted, or it was somehow damaged during the extraction process (perhaps if exposed to oxygen in the air, as is the case with anaerobic bacteria), then it might be rendered inert and incapable of creating disease anew. Practical modifications to his extraction process, such as an extraction environment of carbon dioxide (Martin 1971), would be needed to overcome this.

Koch’s reliance on his visual inspection of microbes to guarantee the purity of his culture (i.e., that microbes looking identical meant that they actually were) is also vulnerable. It is conceivable that two microbes can look very similar and yet be different species. Admittedly, Koch developed staining techniques, some of which are still in use today, to help him differentiate bacterial species (Blevins and Bronze 2010). Another solution might have been to use a ‘selective’ growth medium, which contain ingredients that inhibit the growth of unwanted microorganisms while supporting the growth of the microbe of interest. Alexander Fleming’s first description of penicillin (Fleming 1929) highlighted its virtues in creating a selective medium for isolating a pure culture of *Haemophilus influenzae* (formerly called *Bacillus influenzae* or *Pfeiffer’s bacillus*): “*It sometimes happens that in the human body a pathogenic microbe may be difficult to isolate because it occurs in association with others which grow more profusely and which mask it. If in such a case the first microbe is insensitive to penicillin and the obscuring microbes are sensitive, then by the use of this substance these latter can be inhibited while the former are allowed to develop normally. Such an example occurs in the body, certainly with B. influenza (Pfeiffer) and probably with Bordet’s whooping-cough bacillus and other organisms.*”

Yet another vulnerability of isolation is that it is possible to wholly extract an individual microbe from a colony, yet not completely isolate it from all potential confounders. For example, what if the extracted microbe contained a parasite, perhaps a virus, capable of causing disease? This is not as far-fetched as it might seem: the harmful toxins that cause cholera are only produced if the *Vibrio cholerae* bacterium is infected by a certain ‘bacteriophage’ virus (Nelson et al. 2009). While the virus, by itself, might not directly harm humans, the changed dispositions of the host bacterium most certainly can. To deal with this problem, Koch would have needed some sort of intervention to either neutralise or remove the bacteriophage, or indeed start afresh with new, uncontaminated cultures (Łos et al. 2004).

Finally, a generalised version of Koch’s postulates can provide causal evidence without explicit incorporation of the process of isolation. Indeed, it is successful disease recreation that is paramount for gaining causal *evidence* (a counterfactual), not the details of the methods employed to achieve this (Broadbent 2013). As such, when considering the generation of causal evidence, isolation should not be regarded as a necessary postulate.

### Introducing counter-counterfactuals

Recreating a specific disease in one or more previously healthy subjects, while all control subjects remain healthy, establishes a counterfactual. However, a counterfactual cannot be used to discover the aetiological cause of a disease. It can only *confirm* the aetiological cause of a disease if a singular agent, such as a bacterial isolate, is *sufficient* on its own to cause pathology (and subsequent disease). If multiple factors are necessary to give rise to a particular pathological state, these factors might not be separately isolatable and/or reconstitutable in order to create the disease anew. The only option seems to be to prospectively observe the disease being recreated with all factors in place but in a ‘black box’ (i.e., uncertain) state. This approach would at least avoid some of the vulnerabilities associated with isolating agents before recreating disease. Fortunately, this black box can be peered into if a counterfactual is accompanied by what we might call a *counter-counterfactual*.

To understand what a counter-counterfactual is, it helps to expand the Humean description of a counterfactual mentioned previously: if the first event – *and all upon which it depends –* had not been, the second [event] would have never existed. The clause ‘and all upon which it depends’ is nonessential; adding it to Hume’s description does not change its original meaning nor any of its implications. However, this inserted clause reveals where a counter-counterfactual *acts* (in disease causation, this clause relates to aetiological factors, as represented in Fig. 1). A counter-counterfactual is an action that changes something in the first event that is a necessary component of the second event. It is an example of what Gillies (2016, 2019) describes as an ‘avoidance action’: *sublata causa, tollitur effectus*, which can be translated to ‘if the cause is removed, the effect is taken away.’

To test whether an intervention sufficiently acts as a counter-counterfactual, we must try to change the first event to see if this prevents the second. Obviously, such a test requires a comparison with (or existing knowledge of) what happens in the absence of the counter-counterfactual (i.e., the uninterrupted counterfactual). This is why comparators (or controls) are typically required in such a test. To gain maximum causal information, we should change just one component of the first event to see if this is capable of preventing the second. If it does, we can call this a *minimally sufficient* counter-counterfactual. In contrast, if we prevent every aspect of the first event, we can call this a *total* counter-counterfactual.

In a minimally sufficient counter-counterfactual, we confirm that the changed component of the first event *is necessary* for the occurrence of the second event without preventing any other aspect of the first event. Due to its minimally invasive nature, this is *not* the only information we can gain here. Crucially, knowing the nature of the *action* that successfully prevented the second event gives us previously unavailable information about the *form* of this changed component. In a sense, using a minimally sufficient counter-counterfactual to gain causal information is the inverse of Koch’s isolation process. In order to isolate the suspected causal agent, he would remove *every other* component from his extracted sample. Then, he would introduce the isolated suspected causal agent to a test subject. By comparison, a minimally sufficient counter-counterfactual selectively removes (or neutralises) *only one* suspected causal agent, leaving every other component intact and present. In both cases, we gain information about the single suspected causal agent.

A study that utilises a counter-counterfactual comparison requires a minimum of two actions (interventions): one (the counterfactual intervention) to create the counterfactual conditional and another (the counter-counterfactual intervention) to interfere with the former. When considering disease causation, the counterfactual intervention will act to create the disease while the counter-counterfactual will act to prevent the disease. If we recall that a counterfactual intervention can be thought of as a difference-maker, a counter-counterfactual intervention can be thought of as a *difference-preventer*. In the context of disease causation, counter-counterfactual interventions can therefore be thought of as *disease-preventers*.

**Fig. 2:**
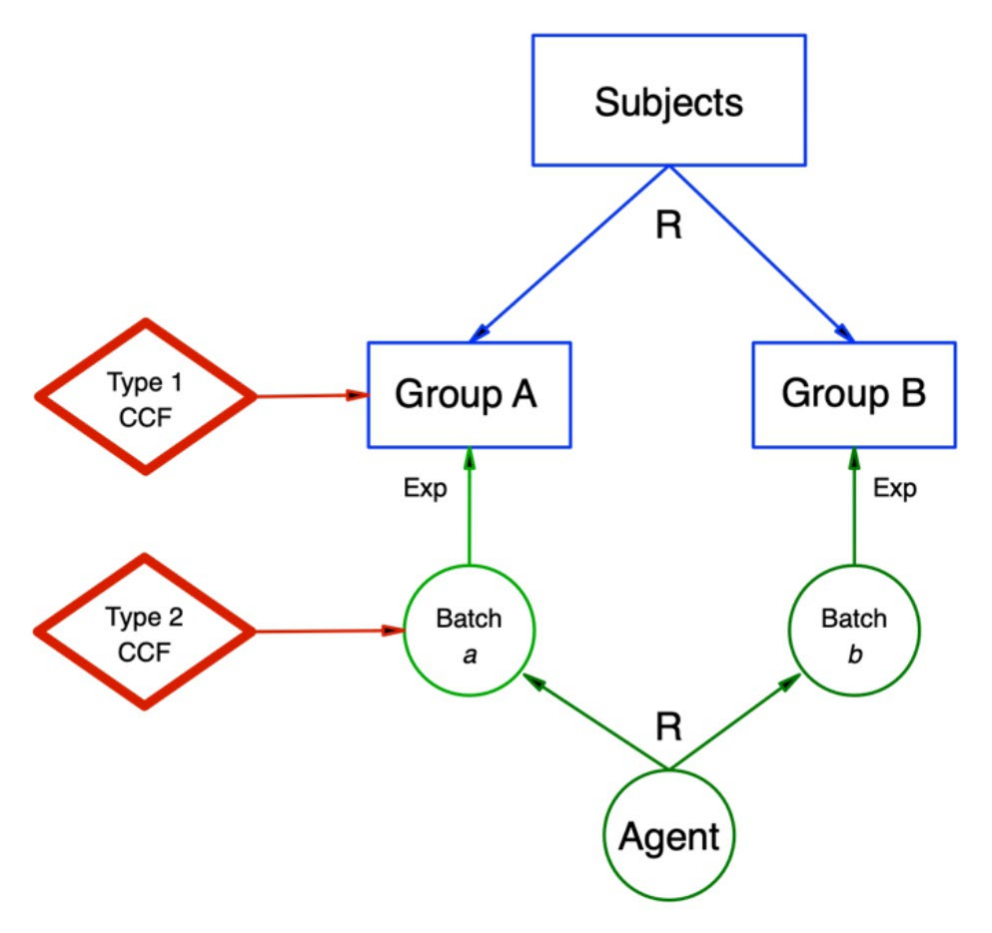
Types of counter-counterfactual interventions

Given this, there are two possible types of disease preventing intervention (Fig. 2): one can be applied *to the subject* in advance of them being exposed to the causal agent; the other can be applied *to the causal agent* in advance of it being introduced to the subject. An obvious example of the former type is a vaccine, which is an intervention applied *to the subject* before they are exposed to a known pathogen. If a vaccine with known structure prevents a disease, we gain useful information about the pathogen. This type of counter-counterfactual can even be applied before the subject technically exists, such as mice deliberately bred with a ‘knockout’ gene; a selectively altered genome designed to study the effects of a single gene being absent. An example of the latter type of counter-counterfactual would be to boil water suspected of making individuals unwell *after drinking it* (drinking the water is the counterfactual conditional here). If individuals drinking the water only after it has been boiled were *not* becoming unwell, we can at least conclude that something in the water was causing the illness.

CCF = counter-counterfactual; R = randomisation; Agent = causal agent.

Experimental design where subjects are exposed to a causal agent, incorporating a counter-counterfactual intervention. Type 1 acts upon one group of subjects, whereas Type 2 acts upon one batch of the causal agent. Each type of counter-counterfactual intervention would be applied within a separate experiment.

In the second example above, the simplest counter-counterfactual intervention that allows us to conclude that something in the water was causing the illness would be to entirely prevent individuals drinking the contaminated water. This is an example of a total counter-counterfactual. In 1854, the English physician, John Snow, did just this. He famously stopped people drinking water from a pump in Soho, London, to demonstrate that the water was the source of a cholera outbreak. While obviously the right thing to do for public health, by completely preventing the first event (the counterfactual conditional of drinking the water) this intervention limited the information available about components of the first event (such as identifying the contaminant within the water) that were necessary for the second event (the development of cholera in people drinking the water). By comparison (and with much hindsight), boiling the water is one intervention that would have *changed* the causal agent while all other aspects of the counterfactual conditional remained intact: the subjects could still have drunk water originating from the contaminated water pump without becoming poorly. This is an example of a minimally sufficient counter-counterfactual. Using this, and with prior knowledge of boiling water, we will have gained additional information about the contaminant *within the water* that was actually responsible for causing the disease: for a start, it was most likely destroyed or rendered inert at 100° C. Additionally, by subsequently attempting to decontaminate the water in a variety of different ways (i.e., attempting other minimal counter-counterfactuals, such as using specific filters or adding a chemical that is harmless to the subjects but which selectively kills known pathogens), even more information could have been gained.

**Fig. 3:**
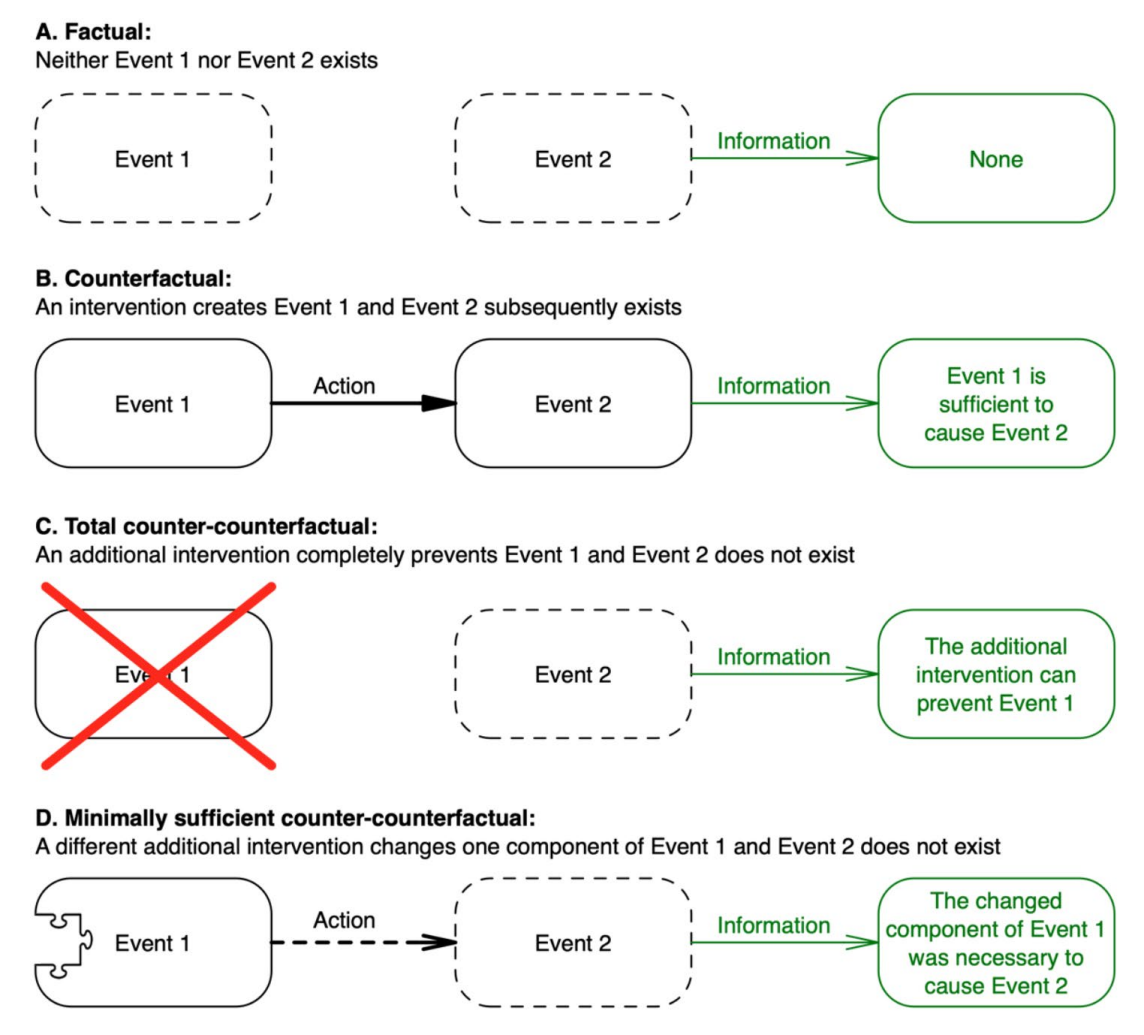
Sequence of investigations to gain information about the cause(s) of an event

It is important to reiterate that the causal information gained by using a counter-counterfactual relies on there being some prior mechanistic knowledge of the counter-counterfactual intervention used (e.g., that a known chemical selectively destroys bacteria or that a known vaccine prevents a disease). Nevertheless, to gain maximum information, the logical sequence of investigation for gaining progressively more information about disease causation is to follow a counterfactual with a total counter-counterfactual, and then a series of minimal counter-counterfactuals that are each sufficient to prevent the second event without completely preventing the first event (Fig. 3).

Applying counter-counterfactuals (disease-preventers) *after* a pathogenic causal agent has been introduced to the test subject will be too late to investigate disease aetiology. Pathogenesis must not have commenced if a counter-counterfactual is to yield information about aetiological factors. This is why counter-counterfactuals cannot be utilised to identify aetiological causes in naturally occurring cases of disease, in which symptoms and signs caused by pathology will already be evident. In such cases, information gained from therapeutic interventions will be largely diagnostic and relate to proximate causes of continuing pathology and consequent patho-physiology (Fig. 1). This diagnostic approach is known as a ‘test of treatment’ (Glasziou et al. 2009).

Although absent from his famed postulates, Koch and his colleagues evidently used counter-counterfactuals when exploring the pathogenic capabilities of microbes. For example, Koch’s mentee, Emil von Behring, began inoculating animals with therapeutic ‘serum’ (an attenuated form of the infectious agent) to see if they remained healthy when later attempting to recreate the disease with the agent itself (Behring & Kitasato 1890). Koch also used a counter-counterfactual to differentiate between the relative effects of the rod-shaped cells and spherical spores of *Bacillus anthracis* (Koch 1876). He would dry a sample of rod-shaped structures with heat before injecting them into a healthy animal to see if they were rendered inert: they were, but the spherical structures were more resilient and not readily neutralised in this way. Koch therefore recognised that neutralising microbial agents in various ways could provide useful information about their form. Indeed, Gillies (2016, 2019) specifically proposes that causal inferences drawn from Koch’s postulates would be enhanced if a further postulate were added: “*It must be shown that if the micro-organisms are prevented from multiplying in the patient’s body, then the patient will not have the disease.*” This clearly describes a counter-counterfactual.

It is worth comparing the respective information gained in terms of disease causation from a counterfactual and a counter-counterfactual. In a hypothetical laboratory investigation to recreate a specific disease, if the disease arose in an individual subject following deliberate exposure to a sample containing a suspected causal agent, evidence is thus gained that the sample is *sufficient* to cause the disease. The evidence for disease sufficiency is even stronger if multiple cases of the disease are reproduced in this way (due to probabilistic reasoning), and stronger still if unexposed control subjects are used to establish a counterfactual conditional (especially when random allocation and blinding to exposure are utilised). However, using such *production actions* to gain causal evidence, one cannot yet make any claims regarding the *necessity* of the sample (or its constituents) for this disease.

Similarly, we can imagine an additional laboratory investigation in which an avoidance action – a preventative intervention such as a vaccine – is applied to an individual subject who is subsequently exposed to a suspected causal agent. In this situation, a continued absence of disease does not provide evidence that the agent *causes* a disease. Furthermore, if a group of subjects are all given the same preventative intervention and none develop the disease following exposure, this still does not provide evidence that the agent causes the disease. However, if yet another group of subjects are all given the same preventative intervention and then exposed to the suspected agent, but this time an additional ‘control’ group are simultaneously exposed *without* the preventative intervention, and controls subsequently develop the disease while none of the pre-treated group do, this now provides compelling evidence that (1) exposure is sufficient to cause the disease and (2) the preventative intervention is capable of negating one or more necessary components of the disease. But (2) is only available because the disease has been successfully recreated in the untreated control subjects. In other words, a counter-counterfactual can only provide causal information in the presence of a counterfactual.

## Part two: evidence for the causal action(s) of a therapeutic intervention

Following the general principles derived from Koch’s postulates (Table 1), an equivalent set of principles can be declared for a therapeutic intervention (Table 2).

**Table 2:** Evidence for causation in a therapeutic intervention

General principle	Evidence for causation
The disease should change in a way that differs significantly from its natural course in subjects to whom the intervention is applied	***Counterfactual***
The disease should follow its natural course in subjects to whom the intervention is not applied	***Comparison***
Neutralising the intervention should prevent or stop its effects	***Counter-counterfactual***

It is, unfortunately, possible that a well-intended intervention harms the recipient. Accordingly, the general principles described in Table 2 are deliberately worded without an assumption of a net therapeutic effect. It is important to allow for this possibility, since causal reasoning is just as essential when considering adverse events that follow intervention. Nevertheless, for the purposes of our discussion, we will assume that the well-intended intervention *is* therapeutic, and we want to find out why this is the case.

### Targeting

The respective *production actions* (Gillies 2016, 2019) of exposure to a pathogen (for disease recreation) and delivery of a treatment (for therapeutic intervention) can be considered equivalent. However, what might be the equivalent of *isolation* when considering therapeutic intervention? As described in part one, isolation involves preparing a suspected causal agent for a difference-making exposure, with the intent being reproduction of a particular disease. Its equivalent must, therefore, be the preparation of an intervention so that it is likely to make a difference to a particular disease, with the intent being that this difference is therapeutic. This preparation can be termed *targeting*.

**Fig. 4:**
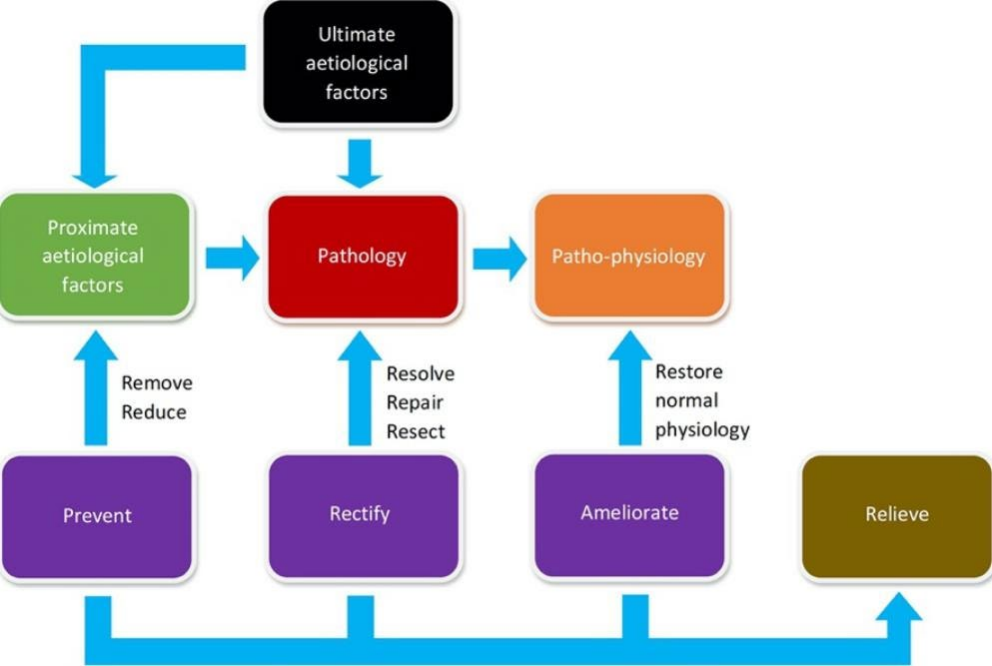
The relationship between therapeutic intervention and the pathological model of disease

To conceive of an intervention that may change the natural course of a particular disease, it helps to have some prior causal knowledge of that disease (e.g., via the sequence of evidence described in part one). At the very least, such information should reveal a potential biological ‘target’ (Goodman & Gerson 2007) which in turn would provide clues to the required *form* of a therapeutic intervention (Evans et al. 2016, 2017). An obvious target would be a necessary causal agent of the disease and/or its descendants (which can be identified via counter-counterfactuals). After all, therapeutic effect can take place via prevention (if the biological target exists within aetiological factors), rectification (pathology), amelioration (patho-physiology), or a combination of these (Fig. 4).

Without any knowledge of a potential biological target, the form of therapeutic interventions must be discovered through trial and error alone. However, even when interventions are discovered through serendipity, some information of a likely biological target is often available to suggest therapeutic potential. For example, as both a physician and bacteriologist, Fleming was already aware of the pathogenic capabilities of staphylococci when he spotted the famous contaminated culture plate.

Given that the priority for intervention is to *change* the course of a disease, rather than to ensure the agent of this change is identified, *targeting* can be more inclusive than its aetiological counterpart, *isolation*. The latter can be investigated later, which is why therapeutic interventions can be used while in a ‘black box’ state, with uncertainty as to their mechanism(s) of action. Furthermore, targeting will incorporate not only the *form* of the intervention, but also the *spatial* route and *timing* of administration (Evans et al. 2016). With the priority being a net therapeutic effect, accuracy (acting upon the target at a certain time) can be more important than specificity (acting only on the target at no other time). Collateral effects can be acceptable if not unduly risky or harmful.

As was argued in part one, a generalised version of Koch’s postulates can provide causal evidence without the process of isolation. As such, targeting should also not be considered necessary, since it too does not provide causal evidence (the reason it does not appear in Table 2). Yet, targeting is often an unavoidable step towards intervention development and optimisation and therefore worth mentioning here.

### Counterfactual

Gaining empirical evidence for causation following intervention with therapeutic intent necessarily begins with a counterfactual conditional (Table 2). In part one, a counterfactual was defined using Hume’s statement: *if the first event had not been, the second would have never existed*. Since the disease must already exist within the subject (otherwise we would not want to intervene), we can interpret the first event of the counterfactual as being the intervention acting on that subject. The second event must therefore equate to any *effects* of that intervention acting on the subject, and in particular any therapeutic effect upon the targeted disease.

Counterfactuals are already well recognised as key to the evaluation of an intervention’s therapeutic efficacy (Mumford and Anjum 2011; Pearl and Mackenzie 2018). Indeed, creating a counterfactual conditional is the basis of a modern randomised controlled trial (RCT). A standard parallel-arm RCT can demonstrate whether an intervention is sufficient to make a difference between two or more groups of subjects. RCTs incorporate association, temporality and spatial proximity; thus, a claim for causation is justified. Association is incorporated because RCTs utilise groups of subjects and results are typically based on comparing the frequency, magnitude and direction of change between groups. Temporality and spatial proximity are provided during application of the intervention, delivered when and where the investigator chooses. The randomisation process distributes known and unknown confounders equally between groups so that (assuming sufficient numbers) we can safely attribute observed effects to the interventions provided for each group. However, the *identity* of the intervention’s causal agent is not revealed as a result of such an RCT. Unless its causal agents are isolated in advance (like Koch’s bacterial cultures), an intervention can remain in a black box state throughout an RCT and stay that way even once its effectiveness has been demonstrated.

### Comparison

Since an intervention is not a spontaneous event, no causal claims can be made until it is delivered. Therefore, evidence of association can only be collected once a counterfactual is underway. A comparison can be made within each subject, before and after receiving an intervention. However, while within-subject comparisons have their virtues, they cannot negate the effects of confounders in the same way that control subjects can (and particularly when random allocation of intervention is utilised). If the intervention has an effect, the counterfactual conditional will be evident as a difference between those who have received it and those who have not. This is an example of evidence for difference-making (Russo & Williamson 2007, 2011). The disease should follow its natural course in subjects to whom the intervention is not applied. Whereas, if the intervention *is* therapeutic, the disease should improve in a way that differs significantly from its natural course in subjects to whom the intervention is applied. Only then should there be specificity of association between groups, which is precisely what an RCT is designed to measure.

### Counter-counterfactual

**Fig. 5:**
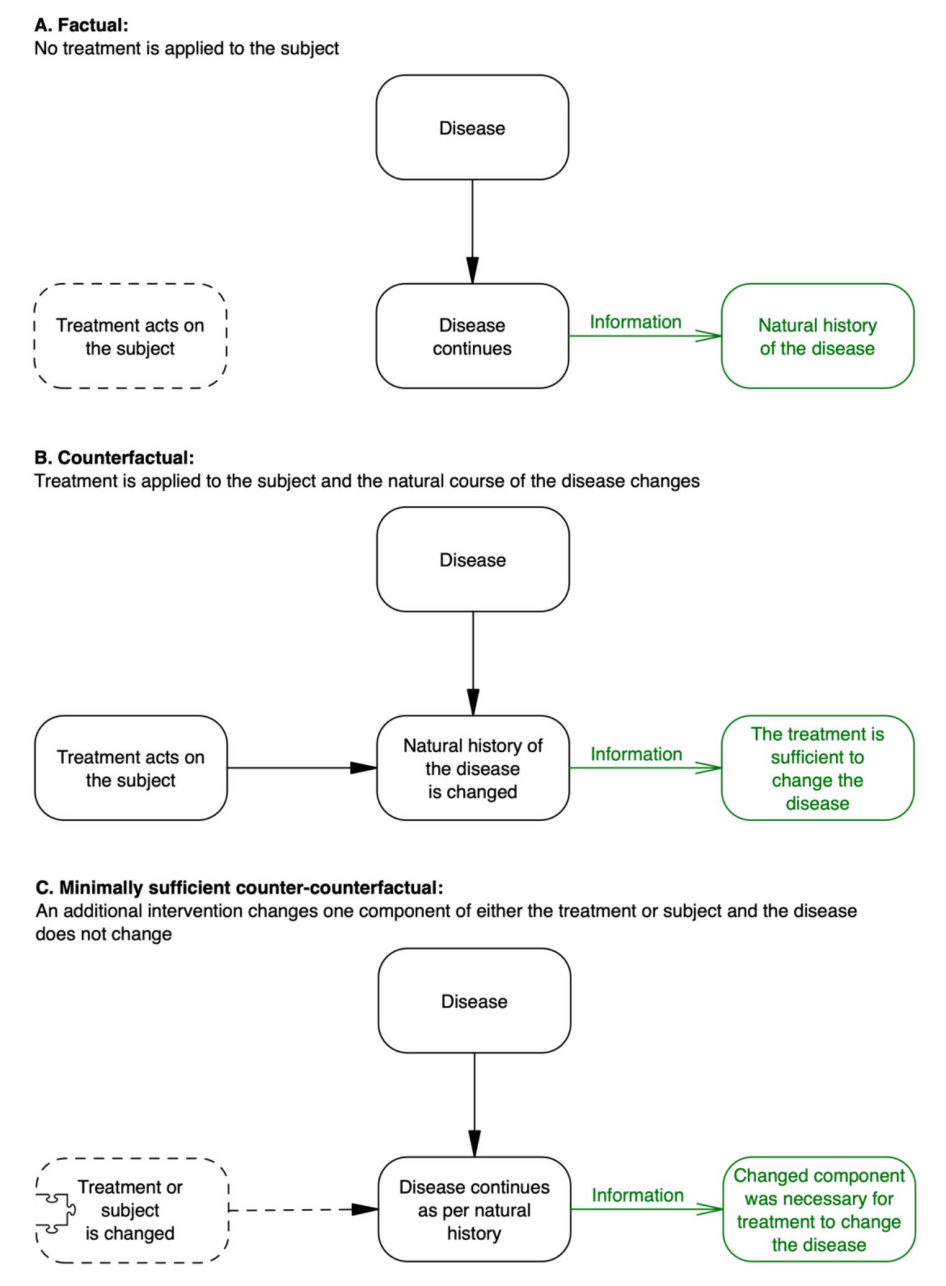
Causal information gained from interventions acting on a disease

What must a counter-counterfactual look like when considering a therapeutic intervention? The earlier nonessential addition to Hume’s statement provides a clue: if the first event – *and all upon which it depends* – had not been, the second [event] would have never existed. With the first event established as the therapeutic intervention *acting on the subject* with the disease, and the second being the *effect* of that intervention acting on the subject, the answer to the above question is: *all upon which the therapeutic intervention acting on the subject depends*. Counter-counterfactuals therefore provide an opportunity to investigate the mechanisms of action of an intervention. If the therapeutic effects of an intervention are prevented or stopped by selectively changing *something*, we can assume that this *something* was necessary for these effects (Fig. 5). In the investigation of the causal actions of a therapeutic intervention, counter-counterfactuals can therefore by thought of as *treatment-preventers*.

Unlike when investigating disease causation, with a therapeutic intervention there is likely to be little additional information gained through a total counter-counterfactual, since this will simply default to a scenario of no intervention at all (scenario A in Fig. 5). Because a disease is already ongoing within a subject, without intervention it will almost certainly continue along its natural course (in fact, an RCT relies upon this assumption). The scenarios in Fig. 5 demonstrate how the causal information that is gained may be interpreted. Interpretation of the counterfactual (scenario B) requires information of the natural history (scenario A). Indeed, scenarios A and B represent two arms of an RCT: scenario B is an active intervention group and A is a no-intervention control group. Without existing knowledge of the natural history (scenario A), one cannot adequately interpret the effect of the intervention on the disease. In turn, interpretation of the counter-counterfactual (scenario C) requires information from the counterfactual (scenario B), so that the relative effects of uninterrupted and interrupted therapeutic intervention can be compared. Hence, the scenarios in Fig. 5 represent a logical sequence of steps for the investigation of mechanisms of action for a therapeutic intervention. Indeed, each scenario can be implemented as one arm of a three-arm RCT.

Just as with counter-counterfactuals related to disease causation (discussed in part one), there are two types of counter-counterfactuals that can be used to prevent treatment effects. As alluded to in Fig. 5, one type can be applied *to the subject* in advance of them being exposed to the intervention; the other can be applied *to the intervention* in advance of it acting on the subject. Indeed, Fig. 2 is equally valid for the use of counter-counterfactuals to study mechanisms of action of a therapeutic intervention (the ‘agent’ in Fig. 2), where a ‘batch’ of the intervention is applied to each group.

An example of a counter-counterfactual that has been applied *to the subject* in advance of them being exposed to a therapeutic intervention is a selective receptor antagonist. These can be used as therapeutic interventions in their own right (e.g. Muñoz & Rosso 2010, Chan et al. 2014, Tricco et al. 2016, do Vale et al. 2019) but are also routinely used to assess the activation of specific biological pathways by other therapeutic interventions, particularly during drug development (e.g. Schäfer et al. 2003, Burghardt et al. 2007, Kuroda et al. 2021). Receptor antagonists prevent a biological response by binding to and blocking a single receptor type, rather than binding to and activating it like an agonist. A novel use of receptor antagonists has been to elucidate some mechanisms of analgesia (Hill 1981), and in particular placebo analgesia. Naloxone (an opioid-specific receptor antagonist) has been administered prior to several interventions known to produce placebo analgesia (Grevert et al. 1983, Amanzio & Benedetti 1999, Benedetti et al. 1999). Acting as a counter-counterfactual intervention, naloxone successfully blocked the analgesic effects of these placebo-inducing interventions, confirming that placebo analgesia acts (at least in part) via opioid pathways. Similarly, proglumide (a cholecystokinin receptor antagonist) has been used as a counter-counterfactual to both enhance opioid-induced placebo analgesic effects and simultaneously prevent hyperalgesia (increased sensitivity to noxious stimuli) caused by ‘nocebo’ interventions (Benedetti 1996, Benedetti et al. 1997). In this example, the information extracted about the mechanisms of action of placebo analgesics relied upon some existing knowledge of the receptor antagonist (e.g., that naloxone selectively blocked opioid receptors). However, the new information gained is valuable, not least because the mechanisms of placebo analgesia are notoriously hard to investigate as the effect can be triggered by a wide range of external interventions, the form of which provide little clue as to the internal biological pathways involved.

An example of a counter-counterfactual applied *to the therapeutic intervention* in advance of it being introduced to the subject is, rather ironically, the basis of a placebo trial. Placebo drugs are best-known, yet placebo surgical procedures (Campbell et al. 2011), acupuncture needles (Streitberger and Kleinhenz 1998), and electrotherapy devices (Buchbinder et al. 2006; Claydon et al. 2011) have also been developed and used in RCTs. Placebo drugs are arguably the simplest to administer within RCTs because every physical aspect of a drug (e.g., colour, size, weight, taste, etc. of the capsule) other than the ‘active’ ingredients can easily be retained. By administering a capsule with inert contents versus a physically identical capsule containing active ingredients, with all other aspects of the RCT being the same for both groups, the difference-making effects of *these active ingredients* can be measured in isolation. Here we can see that, unless the biological target of the active ingredients is already known, placebo RCTs cannot provide information on the mechanism of action of these ingredients. They can only provide information about the *response of the subject* to the active ingredients. If these ingredients remain unidentified, mechanistic inferences are prevented. Nevertheless, it is worth noting that, where placebo interventions are difficult to implement (e.g., physical interventions such as exercise), then a counter-counterfactual applied to the subject, as against the intervention, should provide valuable causal information. For example, naloxone could be used to test whether opioids are involved in the analgesic effects of aerobic exercise within a trial of exercise, versus exercise plus naloxone, versus no exercise. If opioids were primarily responsible for any such effect, then naloxone should reduce exercise-induced analgesia towards that reported by the control subjects.

## Summary

Robert Koch’s logic for causal experimental methodology was second to none. Matched only by the meticulous nature of his laboratory methods, many of which he invented, he was far ahead of his time. He is regarded as the father of bacteriology, but his pioneering work in the investigation of disease causation should be recognised and utilised well beyond this field. Many lessons can still be learned from his work and hopefully some of these have been passed on within this paper.

**Fig. 6:**
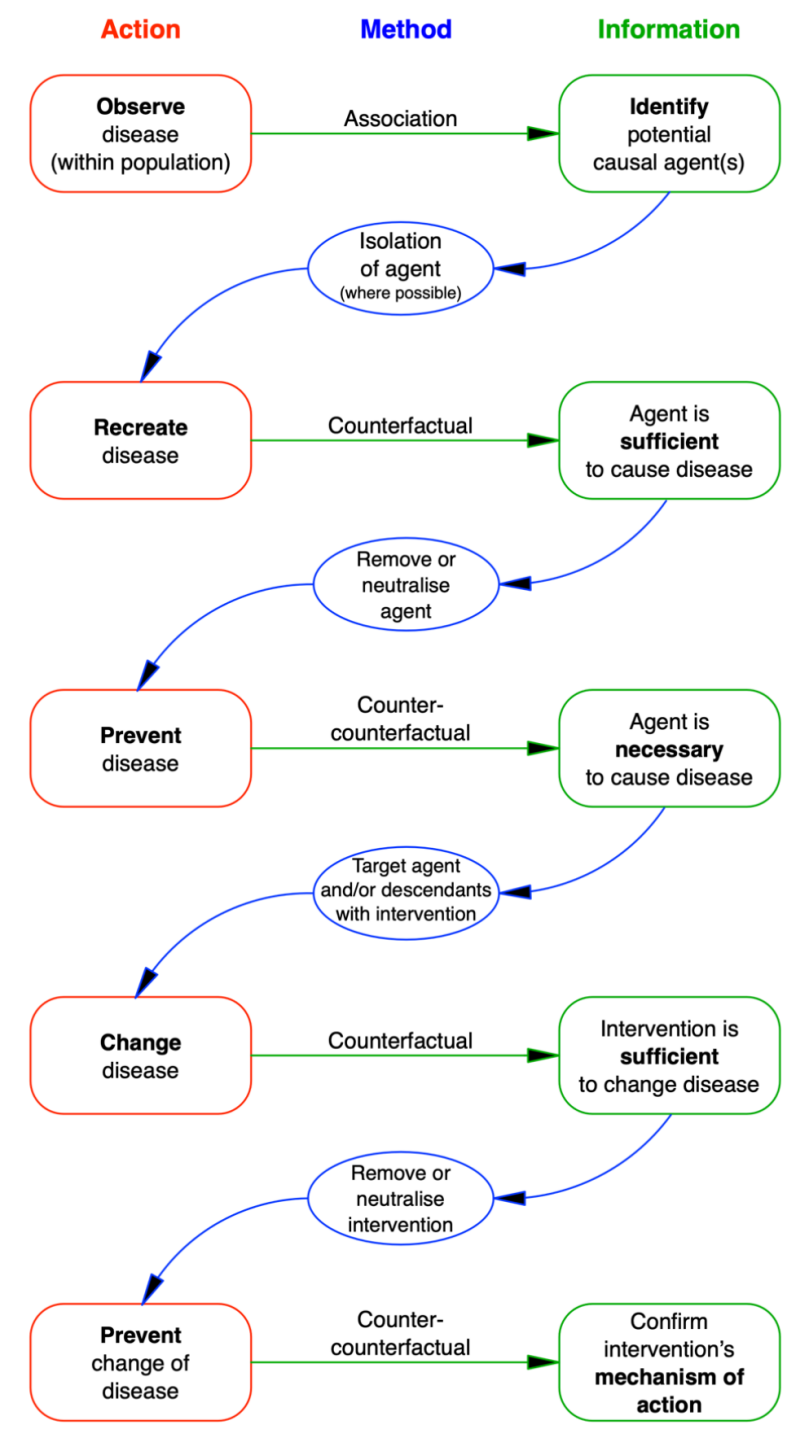
Sequences of evidence

Examining Koch’s postulates, we can see that causes of a disease can be confirmed through a logical sequence of evidence: association between individuals with and without a disease; isolation of suspected causal agents; and the creation of a counterfactual (demonstrating that an agent is sufficient to reproduce the disease anew). An argument is presented here that counter-counterfactuals (disease-preventers) are a useful addition for identifying necessary causes of a disease, and sometimes must be used in place of isolation which is not always possible. In addition, creating a counterfactual (changing the natural course of a disease), comparisons between subjects in receipt of treatment versus those who are not (typically within an RCT), and counter-counterfactuals (treatment-preventers) comprise a logical sequence of causal evidence for a therapeutic intervention, including quantification of its effects and identification of its mechanisms of action. These sequences can be linked together to form a coherent programme of investigation (Fig. 6). As Russo & Williamson (2007, 2011) argue, causal evidence requires both evidence of difference-making and evidence of mechanisms. In the sequences of evidence described here, both evidence of difference-making (counterfactuals, from disease recreation or RCTs) and evidence of mechanisms (counter-counterfactuals, providing information about disease aetiology and mechanisms of action of intervention) are incorporated.

This paper formally introduces counter-counterfactuals, which appear to have been used, perhaps intuitively, since the time of Koch and possibly earlier. These are valuable research tools that, with prior knowledge of their mechanistic action, can be leveraged to gain new causal information about necessary components of both disease aetiology and the mechanisms of action underlying even complex therapeutic interventions (such as placebo effects). Yet, until now they have not been formally recognised or described as a distinct species of causal investigation. Hopefully, this paper will encourage researchers to include them in their investigations and help clinicians and patients recognise their value.
